# Parental control and adolescent internet addiction: the moderating effect of parent-child relationships

**DOI:** 10.3389/fpubh.2023.1190534

**Published:** 2023-05-25

**Authors:** Xiaoqin Zhu, Chen Deng, Wanyue Bai

**Affiliations:** ^1^Department of Applied Social Sciences, The Hong Kong Polytechnic University, Kowloon, Hong Kong SAR, China; ^2^Faculty of Business Accounting and Finance, The Hong Kong Polytechnic University, Kowloon, Hong Kong SAR, China

**Keywords:** psychological control, behavioral control, maternal, paternal, Chinese adolescents

## Abstract

**Introduction:**

The present study investigated how maternal and paternal controls, including behavioral and psychological controls, predict adolescent Internet addiction, as well as the potential moderating effects of adolescent gender and parent-child relationships on the predictions.

**Methods:**

Data were collected from 1,974 Chinese adolescents (age range = 14–22; mean = 16.47; SD = 0.87; 1,099 girls) in Guizhou Province, mainland China in November 2021. Internet addiction was measured using the ten-item Internet Addiction Test developed by Kimberly Young, and Parental control and parent-child relationships were measured by the respective subscales derived from the validated Chinese Parent-Child Subsystem Quality Scale.

**Results:**

Hierarchical regression analyses revealed that after the covariates were statistically controlled, both parents' behavioral controls showed significant negative predictions on adolescent Internet addiction, while their psychological control tended to positively predict Internet addiction among adolescents, although the effect was only marginal. In addition, the impacts of maternal and paternal controls were equal, and such impacts did not vary between sons and daughters. While adolescent gender was not a significant moderator, the parent-child relationship quality significantly moderated the effects of paternal behavioral control, paternal psychological control, and maternal psychological control on adolescents' Internet addiction. Specifically, the prediction of paternal behavioral control was stronger while the effect of paternal and maternal psychological control was weaker among adolescents with a positive father-child relationship than those with a moderate or poor father-child relationship.

**Discussion:**

These findings indicate the protective function of parents' behavioral control and the negative impact of psychological control on the development of adolescent Internet addiction. Further, a positive relationship between the father and the adolescent can strengthen the positive effect of paternal behavioral control and mitigate the negative effects of both parents' psychological controls.

## 1. Introduction

Nowadays, the use of the Internet has become indispensable. However, Internet addiction, also known as “problematic/pathological Internet use” or “compulsive Internet use”, has emerged as a significant public health concern worldwide. Internet addiction is characterized by preoccupation, desire, impaired control, difficulty to quit, and disregard of negative consequences in different Internet activities, such as online games, social network services, pornographic sites, video collection, and excessive online shopping (1). Although Internet addiction might be multidimensional and characterized by different neurobiological underpinnings and subtypes, such as cybersexual addiction, information overload, and social network addiction, based on specific addictive behaviors in online settings, it has become increasingly prevalent, especially among adolescents (1, 2).

Adolescents are the most vulnerable to developing Internet addiction in the information age since they face considerable developmental challenges in different domains, including social, emotional, academic, and familial, and often lack effective coping strategies and self-regulation ability (3, 4). Despite the adoption of different assessment tools and criteria on adolescent Internet addiction, a growing body of reports has consistently revealed a relatively high prevalence of Internet addiction among adolescents (5). In Chinese societies, the prevalence rates of Internet addiction among different adolescent samples have been found to be over 20% (6, 7). The issue has drawn much attention because of its close association with other behavioral problems, mental health issues, and social and emotional dysfunctions (4, 8, 9). For instance, a systematic review showed that individuals with Internet addiction are more prone to developing symptoms such as depression, isolation, mental distress, and even suicidal ideations (1).

It was reported that adolescents tend to deal with negative emotions, distress, and frustrations experienced in the real social world through (over)using the Internet to alleviate negative feelings and temporarily escape from real-world troubles (4, 6). As such, understanding the risk and protective social factors that may increase or reduce the likelihood of developing Internet addiction among adolescents is pivotal for effective prevention and intervention. Compared to other social settings, the family represents the most immediate and influential environment where adolescents are socialized. It has been found that family factors, such as parenting practices and relational qualities, significantly impact adolescents' social and behavioral functioning, including Internet addiction (10, 11). Specifically, parental control, as one of the focuses in parenting studies, has been closely associated with Internet addiction in adolescents (12–14).

Barber et al. (15) distinguished between parental behavioral control and psychological control, conceptually clarifying the construct of parental control. Behavioral control refers to parents' efforts to explicitly regulate, monitor, and manage the child's behavior. Moreover, it is considered a protective factor against the child's emotional distress and behavioral problems when such parental supervision is appropriate to the child's age. Prior studies indicated that parental behavioral control is positively associated with adolescents' individual competence, self-discipline, and school performance while negatively associated with adolescents' emotional and behavioral problems (10). In particular, parental behavioral control sets clear rules, helping parents monitor and regulate their children's Internet activities, which can reduce the likelihood of children's misuse (and overuse) of the Internet. Indeed, different empirical studies reported significant relationships between parental behavioral control and adolescents' healthy Internet use (13).

On the other hand, psychological control refers to manipulative, coercive, intrusive, and disrespect strategies used by parents on their children, such as invalidation, emotional blackmail, guilt induction, shaming, unfavorable comparison, or love withdrawal (15, 16). In contrast to behavioral control, which implies due parental authority and discipline, psychological control is likely to exert undue control over the child's thoughts and feelings, violating the child's self-identity, self-worth, and autonomy. As such, it increases the risk of maladjustment, dysfunctional copying mechanisms, and problematic behaviors, such as Internet addiction (15, 17, 18). Empirically, the deleterious effects of parental psychological control on adolescents' development have been demonstrated across cultures (19, 20), such as excessive Internet use as a dysfunctional means of meeting their psychological needs (6). Indeed, psychological control has displayed a significant positive association with adolescent Internet addiction (11, 13, 21).

Despite the general consensus that parental behavioral control is beneficial for adolescent development, while psychological control is detrimental to a child's healthy functioning, a few unaddressed issues warrant more scholarly attention. Particularly, there is a need to portray a more holistic picture of how parental control is related to adolescent Internet addiction by differentiating paternal and maternal impacts. Historically, fathers are relatively underrepresented in research linking parenting characteristics and adolescent development (22). This might be because mothers, more than fathers, are generally the primary caregivers as reflected by more mother-child daily interactions and mothers' greater emotional support, responsiveness, and overall sense of responsibility toward the child (23). Nevertheless, increasing evidence has revealed that fathers also play an important role in shaping children's development (24, 25).

Most previous studies focused either on maternal parenting or overall parenting characteristics, failing to uncover the distinctions between maternal and paternal impacts on adolescents. Studies which distinguish between fathers' and mothers' roles reported inconclusive findings. Some findings reported stronger maternal impacts (26), some found similar maternal and paternal influences (27, 28), and others revealed stronger paternal impacts (29, 30). With specific reference to adolescent Internet addiction, the results are quite equivocal. For example, in Giles and Price's (31) study, only maternal psychological control showed a positive prediction on adolescents' problematic computer use. Similarly, Shek et al. (11) revealed that only maternal psychological control was a significant predictor of adolescent Internet addiction. However, the study showed that paternal, instead of maternal behavioral control, significantly accounted for variance in adolescents' Internet addiction. In contrast, Lansford et al. (30) found that it was paternal psychological control that exhibited a significant effect on the child's maladjustment; and there was no difference in the effect of paternal and maternal behavioral controls. These seemingly inconsistent findings suggest that the effects of fathers and mothers may differ from each other in different ways when it comes to behavioral and psychological control. However, very few studies have explored this possibility comprehensively. In addition, moderating mechanisms that may alter the way paternal and maternal control affect the child have been largely overlooked.

First, boys and girls may be affected by parental control differently. Adolescent boys may be more vulnerable to negative parenting practices, such as punishment, coercion, and over-controlling (32, 33). This may be related to different gender role expectations ascribed to boys and girls. While girls are socialized to be more caring and family-oriented, which make them more receptive and compliant to negative parenting, boys are encouraged to be more independent and assertive, which make them more sensitive to parents' restriction and control (34, 35). This is affirmed by findings that boys are more likely to report unfavorable parenting characteristics (36, 37). Nevertheless, Lansford et al. (30) found that parental control had a stronger influence on externalizing behaviors of girls than on boys. Shek et al. (11) concluded that parental influence on adolescent Internet addiction did not depend on child gender. The inconsistent findings call for more nuanced investigations to have a better understanding of paternal versus maternal impacts on adolescent Internet addiction with reference to child gender.

Second, the quality of parent-child relationship, a vital aspect of family processes (38), may have implications on parental impacts on adolescent Internet addiction. This topic has not been explored in previous studies. Attachment theory (39) suggests that a positive parent-child relationship, characterized by bonding and satisfaction, can provide children with a secure attachment to their parents, creating trust and a secure environment for effective socialization and communication of children's emotions and difficulties. Without it, even adaptive parenting behavior may negatively influence child development (40). A protective parent-child relationship may further enhance the impacts of positive parenting (e.g., behavioral control) while buffering against the negative effects of dysfunctional parenting (e.g., psychological control). Furthermore, emotional security theory (41) provides that positive parent-child relationships foster children's emotional security, enabling them to take others' perspective and appraise their parents' socialization strategies more positively. For instance, they may interpret psychological control as a parental concern or involvement, rather than disrespect or rejection (42). As such, adolescents are more likely to internalize parental control and demands into intrinsic motivation and beliefs, thus experiencing less reluctance and distress (43). Some empirical studies have found that a good relationship with a parent reduced or even reversed the adverse impacts of dysfunctional parenting [e.g., (44–46)].

In addition, the above-mentioned moderating effect may extend to the other parent according to family systems theory, which holds that different subsystems within a family affect each other (47). A positive relationship with one parent may have a spillover effect that evokes acceptance or facilitates the internalization of the other parent's regulations and demands. It is also likely that a good relationship with one parent may compensate for the negative feelings caused by the other parent's unfavorable parenting practices. This type of compensatory process has been tested in terms of heightened interaction with one parent in the absence (or reduction) of another parent's involvement (46). Murray et al. (48) also found that a good father-child relationship buffered against maternal psychological control's negative influence on the child. In summary, theories and findings suggest that the association between one parent's control and adolescent Internet addiction may be moderated by the quality of relationship with either parent. Nevertheless, no research has empirically tested such a proposition.

**Figure 1:**
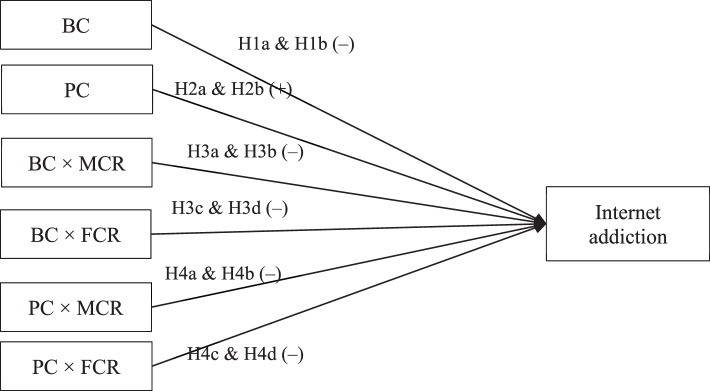
The conceptual framework of the association between parental control (behavioral and psychological control) and adolescents' Internet addiction as well as the moderating effect of parent–child relationships. The effect of maternal and paternal control was tested separately. Control variables are not included for parsimony. BC, behavioral control; PC, psychological control; MCR, mother–child relationship; FCR, father–child relationship.

To address the research gaps in the extant literature, the current study aimed to investigate how maternal and paternal controls (behavioral and psychological control) would be (differently) associated with adolescent Internet addiction, as well as the potential moderating effects of adolescent gender and parent-child relationships. First, both maternal and paternal behavioral controls were expected to negatively predict adolescent Internet addiction (Hypothesis 1a and Hypothesis 1b, respectively), while both parents' psychological controls would positively predict adolescent Internet addiction (Hypothesis 2a and Hypothesis 2b). The magnitude of maternal and paternal impacts was also compared. Given the previous inconsistent findings, the differences were explored without making any specific hypotheses. Second, the moderating effect of adolescent gender was examined, likewise without making specific hypotheses. Third, based on the discussions on the protective effect of high-quality parent-child relationships, it was hypothesized that the prediction of maternal and paternal behavioral control would be moderated (i.e., enhanced) by better mother-child (Hypothesis 3a and Hypothesis 3b, respectively) and father-child relationships (Hypothesis 3c and Hypothesis 3d, respectively). Meanwhile, the prediction of maternal and paternal psychological controls was expected to be moderated (i.e., mitigated) by better mother-child (Hypothesis 4a and Hypothesis 4b, respectively) and father-child relationships (Hypothesis 4c and Hypothesis 4d, respectively) as well. Figure 1 presents the conceptual framework.

## 2. Methods

### 2.1. Participants and procedures

The present study utilized survey data collected in November 2021 from 1,974 adolescents aged 14 to 22 (mean = 16.47, SD = 0.87) in grades 10 and 11 in two high schools in Duyun, Guizhou Province, mainland China. Grade 12 students were not included because they were fully engaged in preparing for the college entrance examination, and the schools did not wish to disrupt their study. All students in the two grades were invited to complete an online survey containing measures on parental factors (e.g., parental control) and developmental outcomes (e.g., Internet addiction) in school computer rooms by class during class hours. The head teacher distributed the survey link to students and reminded them to respond to all questions in the survey based on their own feelings and perceptions. Students read the instructions and information sheet explaining the research objectives and key principles (e.g., anonymity, no-harm, and confidentiality). After the students gave their consent, they were directed to the survey questions. When there were questions left unanswered, remind information directed participants to the unanswered question, allowing them to provide their responses. Thus, the final data set did not have missing values. Following common practice in online survey, two attention checks were included (e.g., “This is an attention check, please choose ‘strongly agree”'). Furthermore, each participant's completion time was recorded to help secure valid responses (49). As a result, we excluded 97 cases whose response durations were shorter or longer than three standard deviations from the mean completion time and/or whose answers to either of the attention checks were incorrect, resulting in the final working sample (*N* = 1,974). Among the participants, 55.67% (*n* = 1,099) were girls. Most of them (*n* = 1,527, 77.36%) indicated that their parents were in their first marriage (i.e., intact family). A total of 290 (14.69%) adolescents reported family dependence on governmental welfare (i.e., poor families). In addition, 485 (24.57%) were the only child (i.e., having no siblings) in the family.

Ethical approval was obtained from the Institutional Review Board (HSEARS20210526004) in the authors' university before data collection. All related parties (i.e., schools, students, and their parents) were informed about the research objectives and principles (e.g., voluntary participation, free withdrawal, anonymity, and confidentiality) and provided their consent.

### 2.2. Measures

*Internet addiction* was measured using the ten-item Internet Addiction Test developed by Kimberly Young (50, 51). The Chinese version utilized in the present study was translated and validated by Shek and colleagues (52). The respondents indicated whether (“1 = Yes”; “0 = No”) they had experienced the ten listed typical symptoms of Internet addiction in the past year (e.g., “Have you repeatedly made unsuccessful efforts to control, cut back, or stop Internet use?” and “Do you feel preoccupied with the Internet or online services and think about it while offline?”). The scale demonstrated a unidimensional structure in the present study, and Cronbach's α and McDonald's ω values (i.e., 0.76) indicated adequate internal consistency of the scale (see Supplementary Table S1). In this study, the Internet Addiction Test score was treated as a continuous variable, consistent with prior research (4, 11). Specifically, adolescents' Internet addiction scores were indexed by the total score across the items, which theoretically range between 0 and 10, with a higher score suggesting a higher level of Internet addiction.

*Parental behavioral control and psychological control* were measured by the respective subscales derived from the validated Chinese Parent-Child Subsystem Quality Scale that has been frequently used to measure Chinese adolescents' perceptions of parental factors (53, 54). Each parent's behavioral control was assessed using seven items (e.g., “My father/mother asked me about what I did after school” and “My father/mother actively understands my afterschool activities”), and psychological control was measured using four items (e.g., “My father/mother often wants to change my mind or feelings for things” and “My father/mother values his/her thoughts and overlooks mine”). The adolescents were instructed to rate each parent's behavioral control and psychological control separately using a four-point Likert scale ranging from 1 (i.e., strongly disagree) to 4 (i.e., strongly agree). The two-factor structure (behavioral and psychological controls) fit the data well in both paternal and maternal subscales, and the Cronbach's α and McDonald's ω estimates of all subscales exceeded 0.90 (see Supplementary Table S1). The average score of each subscale was calculated.

*Parent-child relationships* were measured using the respective paternal and maternal subscales in the aforementioned Parent-Child Subsystem Quality Scale (53). Each subscale was comprised of six items (e.g., “I proactively share my feelings with my father/mother” and “my father's/mother's discipline of me is beneficial to me”) that were rated on a four-point scale (1 = “strongly disagree” to 4 = “strongly agree”). Cronbach's αs and McDonald's ωs were all above 0.90 in the present study (see Supplementary Table S1).

*Covariates* included age, gender, existence of siblings, family economic condition, family intactness, and whether the participants have sibling(s). These covariates have been commonly measured in previous youth studies focusing on parental influence on adolescent development (55). A family's dependence on governmental welfare was considered an indicator of poor family economic condition. For family intactness, whether the first marriage subsists was considered an indicator. Conversely, other martial statuses, such as separated, divorced, or re-married, indicated a non-intact family.

### 2.3. Data analysis

Data analyses were performed using SPSS28.0. Reliability analyses were first conducted, followed by descriptive and correlational analyses. Thereafter, several hierarchical regression analyses were performed to examine the main effects of paternal or maternal control and the moderating effects of child gender and the quality of a parent-child relationship. Covariates were included in the regression models at the first step, maternal/paternal behavioral and psychological control at the second step, and potential moderators (i.e., adolescent gender, parent-child relationships) as well their interactions with parental control at the third and fourth steps, respectively. If adolescent gender would have significant interactive effects with parental control (i.e., moderating effect), simple slope analyses would be further conducted to understand the predictions of parental control on Internet addiction among boys compared to girls. In addition, the moderating effects of parent-child relationships on adolescent boys and girls would be investigated separately. If adolescent gender would not have significant moderating effects, the two gender groups would be combined (i.e., using the whole sample) and adolescent gender would be treated as a covariate for analyses. Using bias-corrected bootstrap, 95% confidence intervals were computed for regression coefficients using 2,000 re-samplings.

## 3. Results

Both parents' behavioral controls and their relationships with adolescents were negatively correlated with adolescent Internet addiction (*r* ranged between −0.20 and −0.10, *p*s < 0.001), while parental psychological control showed no significant correlations with Internet addiction (see Supplementary Table S1). Adolescent gender was not significantly correlated with Internet addiction. Furthermore, adolescent gender did not have significant interactions with maternal or paternal behavioral control or psychological control (*t*s = 0.61–1.31, *p*s = 0.19–0.54) on Internet addiction in the regression models. Thus, all formal analyses were based on the whole sample, with gender included in the regressions as one of the covariates.

Results of the hierarchical regression examining the main effects of parental control and its interactions with parent-adolescent relationships are presented in Table 1 (maternal control) and Table 2 (paternal control). For all regressions, the values of variance inflation factor (VIF) were below 3.0. Considering the cutoff point of 5.0 where the issue of multicollinearity becomes a major concern (56), the current results indicated that multicollinearity was not a major problem in the present study.

**Table 1:** Hierarchical regression analysis for the predictions of maternal control and its interactions with parent–child relationship on adolescent internet addiction.

**Model**	**Predictor**	***B***	***BC 95% CI***		***SE***	***Beta***	***t***	***Cohen's f^2^***	***VIF***	***F* change**	***R^2^* change**
			***Lower***	***Upper***							
1	Age	−0.17	−0.28	−0.05	0.06	−0.07	−2.90^**^	0.004	1.03	2.13^∧^	0.01
	Gender^a^	0.11	−0.09	0.31	0.10	0.02	1.04	0.00	1.02		
	FEC^b^	−0.08	−0.36	0.20	0.14	−0.01	−0.55	0.00	1.04		
	FI^c^	−0.01	−0.24	0.23	0.12	0.00	−0.06	0.00	1.03		
	HS^d^	−0.09	−0.32	0.15	0.12	−0.02	−0.73	0.00	1.07		
2	Age	−0.19	−0.30	−0.08	0.06	−0.07	−3.26^**^	0.01	1.03	18.52^***^	0.02
	Gender^a^	0.16	−0.04	0.36	0.10	0.04	1.57	0.00	1.07		
	FEC^b^	−0.03	−0.31	0.25	0.14	−0.01	−0.23	0.00	1.05		
	FI^c^	0.05	−0.19	0.28	0.12	0.01	0.40	0.00	1.04		
	HS^d^	−0.05	−0.29	0.18	0.12	−0.01	−0.45	0.00	1.08		
	MBC	−0.48	−0.64	−0.32	0.08	−0.14	−5.92^***^	0.02	1.06		
	MPC	0.15	0.04	0.27	0.06	0.06	2.56^*^	0.003	1.10		
3	Age	−0.18	−0.29	−0.06	0.06	−0.07	−3.08^**^	0.005	1.03	26.87^***^	0.03
	Gender^a^	0.11	−0.09	0.31	0.10	0.02	1.06	0.00	1.08		
	FEC^b^	−0.05	−0.33	0.23	0.14	−0.01	−0.35	0.00	1.05		
	FI^c^	0.17	−0.07	0.40	0.12	0.03	1.38	0.00	1.06		
	HS^d^	−0.07	−0.30	0.16	0.12	−0.01	−0.61	0.00	1.08		
	MBC	−0.34	−0.58	−0.10	0.12	−0.10	−2.79^**^	0.004	2.47		
	MPC	0.14	0.02	0.26	0.06	0.05	2.36^*^	0.003	1.12		
	MCR	0.31	0.04	0.58	0.14	0.08	2.29^*^	0.003	2.66		
	FCR	−0.68	−0.86	−0.50	0.09	−0.20	−7.33^***^	0.03	1.57		
4	Age	−0.17	−0.28	−0.06	0.06	−0.07	−2.97^**^	0.004	1.04	3.41^**^	0.01
	Gender^a^	0.08	−0.12	0.28	0.10	0.02	0.76	0.00	1.09		
	FEC^b^	−0.06	−0.34	0.21	0.14	−0.01	−0.44	0.00	1.05		
	FI^c^	0.16	−0.08	0.39	0.12	0.03	1.32	0.00	1.06		
	HS^d^	−0.07	−0.31	0.16	0.12	−0.01	−0.63	0.00	1.08		
	MBC	−0.38	−0.61	−0.14	0.12	−0.11	−3.09^**^	0.005	2.48		
	MPC	0.20	0.07	0.32	0.06	0.08	3.09	0.00	1.30		
	MCR	0.29	0.02	0.56	0.14	0.08	2.08^*^	0.002	2.70		
	FCR	−0.66	−0.84	−0.47	0.09	−0.19	−6.99^***^	0.02	1.60		
	MBC × MCR	−0.03	−0.12	0.06	0.05	−0.02	−0.56	0.00	2.10		
	MBC × FCR	−0.01	−0.12	0.09	0.05	−0.01	−0.24	0.00	1.98		
	MPC × MCR	−0.05	−0.16	0.06	0.05	−0.03	−0.88	0.00	2.00		
	MPC × FCR	−0.10	−0.20	0.01	0.05	−0.05	−1.81^∧^	0.002	1.80		

^a^1 = male, 2 = female; ^b^FEC = family economic condition (1 = poor, 2 = not poor); ^c^FI = family intactness (1 = not intact, 2 = intact); ^d^HS = Having siblings (1 = yes, 2 = no); BC, bias corrected; CI, confidence interval; VIF, variance inflation factor; MBC, mothers' behavioral control; MPC, mothers' psychological control; MCR, mother–child relationship; FCR, father–child relationship. ^∧^*p* < 0.10; ^*^p < 0.05; ^**^p < 0.01; ^***^p < 0.001.

**Table 2:** Hierarchical regression analysis for the predictions of paternal control and its interactions with parent–child relationship on adolescent internet addiction.

**Model**	**Predictor**	***B***	***BC 95% CI***		***SE***	***Beta***	***t***	***Cohen's f^2^***	***VIF***	***F* change**	***R^2^* change**
			***Lower***	***Upper***							
1	Age	−0.17	−0.28	−0.05	0.06	−0.07	−2.90^**^	0.004	1.03	2.13^∧^	0.01
	Gender^a^	0.11	−0.09	0.31	0.10	0.02	1.04	0.00	1.02		
	FEC^b^	−0.08	−0.36	0.20	0.14	−0.01	−0.55	0.00	1.04		
	FI^c^	−0.01	−0.24	0.23	0.12	0.00	−0.06	0.00	1.03		
	HS^d^	−0.09	−0.32	0.15	0.12	−0.02	−0.73	0.00	1.07		
2	Age	−0.17	−0.29	−0.06	0.06	−0.07	−3.03^**^	0.005	1.03	36.55^***^	0.04
	Gender^a^	0.07	−0.13	0.27	0.10	0.02	0.67	0.00	1.09		
	FEC^b^	−0.08	−0.36	0.20	0.14	−0.01	−0.57	0.00	1.04		
	FI^c^	0.15	−0.09	0.38	0.12	0.03	1.23	0.00	1.05		
	HS^d^	−0.07	−0.30	0.16	0.12	−0.01	−0.62	0.00	1.07		
	FBC	−0.64	−0.78	−0.49	0.07	−0.20	−8.54^***^	0.04	1.11		
	FPC	0.12	0.00	0.24	0.06	0.04	1.88^∧^	0.002	1.15		
3	Age	−0.17	−0.28	−0.06	0.06	−0.07	−2.97^**^	0.004	1.03	8.23^***^	0.01
	Gender^a^	0.08	−0.13	0.28	0.10	0.02	0.73	0.00	1.10		
	FEC^b^	−0.08	−0.35	0.20	0.14	−0.01	−0.55	0.00	1.04		
	FI^c^	0.18	−0.05	0.42	0.12	0.03	1.52	0.00	1.06		
	HS^d^	−0.08	−0.31	0.15	0.12	−0.02	−0.68	0.00	1.07		
	FBC	−0.32	−0.54	−0.11	0.11	−0.10	−2.92^**^	0.004	2.47		
	FPC	0.10	−0.02	0.22	0.06	0.04	1.57	0.00	1.15		
	MCR	0.10	−0.11	0.30	0.10	0.03	0.93	0.00	1.53		
	FCR	−0.49	−0.73	−0.25	0.12	−0.14	−4.01^***^	0.01	2.70		
4	Age	−0.17	−0.28	−0.06	0.06	−0.07	−2.97^**^	0.004	1.04	7.38^***^	0.01
	Gender^a^	0.04	−0.17	0.24	0.10	0.01	0.35	0.00	1.12		
	FEC^b^	−0.09	−0.36	0.19	0.14	−0.01	−0.61	0.00	1.04		
	FI^c^	0.13	−0.10	0.37	0.12	0.03	1.12	0.00	1.07		
	HS^d^	−0.05	−0.28	0.18	0.12	−0.01	−0.39	0.00	1.08		
	FBC	−0.37	−0.59	−0.15	0.11	−0.11	−3.32^***^	0.01	2.50		
	FPC	0.15	0.02	0.29	0.07	0.06	2.28^*^	0.003	1.27		
	MCR	0.21	−0.01	0.43	0.11	0.05	1.84	0.00	1.83		
	FCR	−0.62	−0.87	−0.37	0.13	−0.18	−4.86^***^	0.01	2.99		
	FBC × MCR	0.06	−0.04	0.15	0.05	0.03	1.14	0.00	2.02		
	FBC × FCR	−0.17	−0.26	−0.09	0.04	−0.12	−4.04^***^	0.01	2.55		
	FPC × MCR	0.00	−0.10	0.10	0.05	0.00	−0.04	0.00	2.42		
	FPC × FCR	−0.12	−0.23	−0.01	0.06	−0.07	−2.21^*^	0.002	2.71		

^a^1 = male, 2 = female; ^b^FEC = family economic condition (1 = poor, 2 = not poor); ^c^FI = family intactness (1 = not intact, 2 = intact); ^d^ HS = Having siblings (1 = yes, 2 = no); BC, bias corrected; CI, confidence interval; VIF, variance inflation factor; FBC, fathers' behavioral control; FPC, fathers' psychological control; MCR, mother–child relationship; FCR, father–child relationship. ^∧^*p* < 0.10; ^*^p < 0.05; ^**^p < 0.01; ^***^p < 0.001.

As shown in Table 1, after the covariates were statistically controlled, maternal behavioral control negatively predicted adolescent Internet addiction (*β* = −0.14, *p* < 0.001, *Cohen's f*^2^ = 0.02), while maternal psychological control showed a positive prediction (*β* = 0.06, *p* < 0.05, *Cohen's f*^2^ = 0.003). Similar to maternal influences, paternal behavioral control also served as a negative predictor of adolescent Internet addiction (*β* = −0.20, *p* < 0.001, *Cohen's f*^2^ = 0.04), while paternal psychological control showed a marginally significant positive prediction (*β* = 0.04, *p* = 0.06, *Cohen's f*^2^ = 0.002, see Table 2). These results supported Hypotheses 1a, 1b, 2a, and 2b. Further comparisons between regression coefficients revealed that behavioral control showed a stronger effect than psychological control for both maternal influence (absolute value of *z* = 6.29, *p* < 0.05) and paternal influence (absolute value of *z* = 7.76, *p* < 0.05). There were no significant differences between maternal and paternal effects regarding behavioral control (absolute value of *z* = 1.46, *p* > 0.05) or psychological control (absolute value of *z* = 0.40, *p* > 0.05).

As shown in Table 1, father-child relationships showed a marginally significant interaction with maternal psychological control on adolescent Internet addiction (*β* = −0.05, *p* = 0.07, *Cohen's f*^2^ = 0.002), suggesting that the prediction of maternal psychological control tended to be moderated by father-child relationship (Hypothesis 4c was marginally supported). Analyses of simple slopes (see Table 3) suggested that maternal psychological control tended to have a positive effect on adolescent Internet addiction when father-child relationship was not high (below +1 SD), while such a positive prediction might be reversed (although it was not significant) when adolescents reported positive father-child relationship (+1 SD). No other moderating effects were identified for maternal control's predictions on adolescent Internet addiction.

**Table 3:** Simple slopes of parental control on adolescent internet addiction at different levels of father–child relationship.

**Predictor**	**Father–child relationship**	***B***	***BC 95% CI***		***SE***	***Beta***	***t***	***Cohen's f^2^***	***F* change**	***R^2^* change**
			***Lower***	***Upper***						
Maternal psychological control	Low	0.30	−0.08	0.69	0.19	0.11	1.57	0.01	1.67	0.04
	Middle	0.22	0.06	0.38	0.08	0.07	2.72^**^	0.01	2.94^**^	0.01
	High	−0.14	−0.31	0.04	0.09	−0.08	−1.52	0.01	0.98	0.02
Paternal behavioral control	Low	0.33	−0.11	0.77	0.22	0.10	1.49	0.01	1.63	0.04
	Middle	−0.57	−0.82	−0.32	0.13	−0.12	−4.54^***^	0.02	5.16^***^	0.02
	High	−0.80	−1.20	−0.41	0.20	−0.21	−3.99^***^	0.04	3.27^**^	0.05
Paternal psychological control	Low	0.38	0.01	0.75	0.19	0.14	2.04^*^	0.02	1.97^∧^	0.05
	Middle	0.08	−0.10	0.25	0.09	0.02	0.85	0.001	1.82	0.004
	High	−0.16	−0.34	0.02	0.09	−0.10	−1.70	0.01	1.08	0.02

Control variables are not shown in the table. ^∧^*p* < 0.10; ^*^p < 0.05; ^**^p < 0.01; ^***^p < 0.001.

Regarding the moderating effects on paternal control's associations with adolescent Internet addiction, while a mother-child relationship did not serve as a significant moderator, a father-child relationship demonstrated significant interactions with both paternal behavioral control (*β* = −0.12, *p* < 0.001, *Cohen's f*^2^ = 0.01) and psychological control (*β* = −0.07, *p* < 0.05, *Cohen's f*^2^ = 0.001). Thus, Hypotheses H3d and H4d were supported. Analyses of simple slopes (see Table 3) revealed that the negative prediction effect of paternal behavioral control on adolescent Internet addiction was stronger for adolescents experiencing positive (+1 SD) father-child relationship quality (*β* = −0.21, *p* < 0.001, *Cohen's f*^2^ = 0.04), while the effect was not significant among adolescents reporting negative (−1 SD) father-child relationship quality (*β* = 0.10, *p* = 0.14, *Cohen's f*^2^ = 0.01). Furthermore, paternal psychological control only showed a significant positive effect on adolescent Internet addiction in the condition of negative (−1 SD) father-child relationship (*β* = 0.14, *p* < 0.05, *Cohen's f*^2^ = 0.02). When the father-child relationship was better, the positive prediction of paternal psychological control on adolescent Internet addiction became insignificant. Meanwhile, when it was positive (+1 SD), the prediction became negative (although insignificant).

## 4. Discussion

The first objective of the present study was to examine how parental control predicted adolescents' Internet addiction among Chinese high school students by separating behavioral control and psychological control and differentiating maternal and paternal influences. In general, both maternal and paternal behavioral controls served as protective factors that were negatively associated with adolescent Internet addiction, while both parents' psychological controls were risk factors that increased the likelihood of adolescent Internet addiction. The findings on parental behavioral control are largely consistent with previous conclusions and suggest that behavioral control is one of the positive parenting strategies that help promote healthy behavior among children, including the appropriate use of Internet by setting clear regulations and exercising suitable supervision (13, 14, 57). In addition, the use of behavioral management strategies in parenting seemed more influential than parental psychological control, which confirms previous findings (57, 58).

In contrast, parental psychological control showed marginal positive predictions on adolescent Internet addiction. Similar observations were outlined by Shek and colleagues in their studies on parental influences on the development of Internet addiction among Chinese adolescents in Hong Kong (11, 14). To some extent, these findings echo the previous proposition that psychological control is a dysfunctional parenting practice impairing the child's healthy functioning in different societies (19, 59). Nevertheless, some prior studies reported that parental psychological control insignificantly affects a child's developmental outcomes (54, 55, 57). The weak or insignificant effect of parental psychological control may imply different interpretations of parenting practices in Chinese societies. Specifically, parents' psychological control might be more permissible and acceptable in a collectivistic context, such as in China, as it is used by parents to achieve mainstream socialization goals, in contrast to individualistic contexts where it is perceived negatively (60). Meanwhile, findings based on a global unidimensional measure of psychological control in the present and most prior research may be unable to fully unveil the influence of parental psychological control given the multidimensional nature of psychological control, and different subtypes of psychological control may yield distinct or even contrasted implications (19, 61, 62). Thus, the current findings do not necessarily suggest that the impact of parental psychological control among Chinese adolescents is negligible; however, there is an urgent need to understand operations of individual dimensions in adolescent development.

In the present study, maternal and paternal behavioral and psychological controls showed similar predictive effects on adolescent Internet addiction. Considering that only father-child, not mother-child, relationships functioned as a moderator, it may be assumed that paternal impacts are relatively greater. This observation echoes previous findings that suggest similar paternal and maternal impacts or even greater paternal impact in shaping adolescents' developmental outcomes, including Internet addiction (11, 14, 28, 30). Despite insufficient representation of fathering in previous studies, the role of fathers is significant in the life of adolescents (24, 58). The findings of the present study provide additional empirical evidence for such a claim. Generally, fathers are less devoted to taking care of their children; and adolescents also usually prefer maternal parenting characteristics, as mothers are typically considered interactive, warm, supportive, responsive, responsible, and not controlling (34, 36). Nevertheless, it may be the quality rather than the quantity of a father-child interaction that shapes the child's development. This interpretation is especially relevant in understanding the unique moderating effect of father-child relationships.

Noteworthily, the relationship between a father and an adolescent significantly moderated the effects of maternal psychological control, paternal behavioral control, and paternal psychological control on adolescent Internet addiction. Specifically, the effect of paternal behavioral control was stronger, while the effect of paternal psychological control was weaker among adolescents who reported higher levels of father-child relationship (+1 SD) than among those who perceived moderate or poor father-child relationships. This novel finding suggests that a father's good relationship with an adolescent can enhance the protective function of their positive parenting, such as behavioral control, on the adolescent and can mitigate the harmful impact of their dysfunctional parenting, such as psychological control. Furthermore, such a moderating effect even spilled over from father-child dyad to mother-child dyad such that in the present study, a positive father-child relationship mitigated the adverse influence of maternal psychological control on adolescent Internet addiction. The finding supports the expectation of family systems theory which holds that different subsystems within a family affect one another (47). In the current study, the mother-child dyad can be influenced by the father-child dyad. This is an insightful finding given that very limited research effort has been spent in understanding such a spillover effect.

Scholars previously argued that a parent-child relationship lays “the fundamental platform” on which parental control operates [(63), p. 472]. Initially, a good relationship with parents signifies a child's intimate attachment to the parents, making the child more willing to assume parents' good intention and comply with parents' disciplines (57, 64). Additionally, a high-quality parent-adolescent relationship creates a safe family environment for adolescents to communicate and disclose their negative feelings and distress, lessening the likelihood of turning to Internet as an escape (11, 53, 65). Meanwhile, a good relationship with one parent may compensate for the negative feelings adolescents may experience in unfavorable interactions with the other parent (46). While the present findings support the essential functions of a father-child relationship, the moderating effect of a mother-child relationship was not identified. One possible explanation may be the generally dominant and superior role of fathers in Chinese families, making father-child relationships bear even more salient implications (11, 58). Nevertheless, more studies are needed to further verify and replicate the present findings.

The present study also explored the impact of adolescent gender on Internet addiction, which was found to be insignificant. Despite a considerable number of previous research which reported that boys commonly displayed higher levels of Internet addiction than adolescent girls (4, 11, 57, 66), some studies, including the current one, did not find discrepancies among both genders with regard to Internet addiction (21, 66). The finding that girls tended to report excessive usage of Internet than boys might be due to the possibility that girls have higher problem awareness relative to boys (66). The similar predictive effects of parental control on boys' and girls' Internet addiction found in the present study echo previous findings that yielded comparable parental influences on sons and daughters (11, 12). Nevertheless, there are also findings indicative of distinct parental impacts on boys and girls. For example, Lansford et al. (30) and Shi et al. (67) found stronger impacts of parental factors on developmental outcomes among girls than boys. In contrast, Shek's (68) study indicated that fathering was more influential on sons' (vs. daughters') mental health and behavioral functioning. Given the inconclusive findings, more research must be conducted to explore gender effect.

The present findings have practical implications for developing prevention and intervention programs for adolescent Internet addiction, especially concerning parental involvement and training which were ignored in previous treatment of Internet addiction (69). The significant protective effects of parental behavioral control and the risk effects of parental psychological control on the development of adolescent Internet addiction suggest that parent training programs should highlight the parents' knowledge and skills to monitor and regulate their children's Internet usage actively and correctly. Moreover, given that father-child relationships are likely to serve as a moderator that can strengthen the beneficial effect of fathers' behavioral control and reduce the harmful impact of parental psychological control, elements of promoting children's relationship with fathers (e.g., youth programs building children's communication skills and parent training enhancing paternal involvement in children's daily life) can be meaningfully incorporated in psychological interventions for Internet addiction. Fostering bonding and trust between fathers and children and creating an open and comfortable family environment can be effective strategies to reduce the risk of adolescents' misuse of the Internet and their reliance on the virtual world.

Several limitations of the present study are noted. First, the data on parental factors and adolescent Internet addiction were collected through adolescent self-report, which may inflate shared variance between the predictors and outcomes. However, child self-report of parenting is widely adopted in parenting studies. Many scholars endorse the advantages of this method because the parent report is not necessarily more accurate than the child report, and it is how children interpret (or perceive) parental behaviors that dictate their adjustment and development (70, 71). In addition, evidence also showed that the associations between parenting and adolescent developmental outcomes were not unduly influenced by common method variance (72). Nevertheless, it will be insightful to adopt multi-informant designs in future studies and further investigate the discrepancies between parent and child reports. Second, a global measure of parental psychological control was utilized, which may hinder the detection of unique effects of subtypes of psychological control (62). It is recommended that future studies adopt a differentiated approach to parental psychological control. Third, the present study involved adolescents in two grades in only two high schools in mainland China. To enhance the generalizability of the findings, the adolescent sample should be expanded to include students from other grades, schools, and Chinese communities (e.g., Hong Kong) in future research. Finally, the current study was cross-sectional and quantitative in nature. To shed light on longitudinal effects of parental control on adolescent Internet addiction, as well as the potential moderating effects of parent-child relationships overtime, and to delineate the in-depth information behind the quantitative findings, it is necessary to employ a mixed-method research design which combines longitudinal quantitative design and qualitative research strategies.

## 5. Conclusion

The present study contributes to existing literature by differentiating between paternal and maternal behavioral controls and psychological controls and exploring the moderating role of parent-child relationships. The findings suggest that both parents' behavioral controls are protective factors, while their psychological controls are risk factors in the development of adolescent Internet addiction. In addition, the quality of a father-child relationship is likely to moderate the impact of parental control, enhancing the beneficial effects of fathers' behavioral control while reducing the harmful effects of both fathers' and mothers' psychological control. These findings suggest that it is essential to promote positive parenting and improve the relationship between parents and adolescents to prevent adolescent Internet addiction.

## Data availability statement

The raw data supporting the conclusions of this article will be made available by the authors, without undue reservation.

## Ethics statement

The studies involving human participants were reviewed and approved by Institutional Review Board at The Hong Kong Polytechnic University. Written informed consent to participate in this study was provided by the participants' legal guardian/next of kin.

## Author contributions

XZ designed the research and contributed to all the steps of the work. CD and WB contributed to manuscript drafting. All authors contributed to the article and approved the submitted version.
