# Supplementary material for: Parental control and adolescent internet addiction: the moderating effect of parent-child relationships
Source: Front Public Health. 2023 May 25;11:1190534. doi: 10.3389/fpubh.2023.1190534 (PMC10248257; doi:10.3389/fpubh.2023.1190534)
Supplement: Supplementary file 1 [file Table_1.DOCX]

| Table S1. Reliability, descriptions, and correlations | | | | | | | | | | | | | | | | |
| --- | --- | --- | --- | --- | --- | --- | --- | --- | --- | --- | --- | --- | --- | --- | --- | --- |
| Variable | Reliability and descriptions | | | |  | Correlations | | | | | | | | | | |
|  | α | ω | Mean | SD |  | 1 | 2 | 3 | 4 | 5 | 6 | 7 | 8 | 9 | 10 | 11 |
| 1. Age |  |  | 16.47 | .87 |  | -- |  |  |  |  |  |  |  |  |  |  |
| 1. Gender ^a^ |  |  |  |  |  | -.06^**^ | -- |  |  |  |  |  |  |  |  |  |
| 1. FEC ^b^ |  |  |  |  |  | -.12^***^ | -.06^*^ | -- |  |  |  |  |  |  |  |  |
| 1. FI ^c^ |  |  |  |  |  | -.07^**^ | -.05^*^ | -.01 | -- |  |  |  |  |  |  |  |
| 1. HS ^d^ |  |  |  |  |  | -.09^***^ | -.11^***^ | .16^***^ | -.15^***^ | -- |  |  |  |  |  |  |
| 1. IA | .76 | .76 | 2.26 | 2.22 |  | -.07^**^ | .03 | -.01 | -.004 | -.02 | -- |  |  |  |  |  |
| 1. MBC | .93 | .93 | 3.07 | .63 |  | -.06^**^ | -.01 | .06^**^ | .07^**^ | .08^***^ | -.12^***^ | -- |  |  |  |  |
| 1. MPC | .94 | .94 | 2.35 | .87 |  | .02 | -.21^***^ | .00 | .02 | .08^***^ | .02 | .20^***^ | -- |  |  |  |
| 1. MCR | .90 | .90 | 3.16 | .58 |  | -.05^*^ | .05^*^ | .04 | .08^***^ | .03 | -.10^***^ | .75^***^ | .06^*^ | -- |  |  |
| 1. FBC | .92 | .92 | 2.88 | .69 |  | .01 | -.10^***^ | .01 | .16^***^ | .01 | -.19^***^ | .59^***^ | .14^***^ | .47^***^ | -- |  |
| 1. FPC | .92 | .92 | 2.34 | .84 |  | .03 | -.24^***^ | .01 | .04 | .07^***^ | -.02 | .18^***^ | .71^***^ | .09^***^ | .28^***^ | -- |
| 1. FCR | .90 | .91 | 2.97 | .66 |  | .01 | -.04 | .01 | .16^***^ | -.01 | -.20^***^ | .50^***^ | .05^*^ | .58^***^ | .75^***^ | .15^***^ |
| Note. ^a^ 1=male, 2=female; ^b^ FEC=family economic condition (1=poor, 2=not poor); ^c^ FI=family intactness (1=not intact, 2=intact); ^d^ HS=Having siblings (1=yes, 2=no); IA=internet addiction; MBC = mothers’ behavioral control; MPC=mothers’ psychological control; MCR=mother–child relationship; FBC=fathers’ behavioral control; FPC=fathers’ psychological control; FCR=father–child relationship. ^*^*p* < .05; ^**^*p* < .01; ^***^*p* < .001. | | | | | | | | | | | | | | | | |
